# Innovation Diffusion: The Influence of Social Media Affordances on Complexity Reduction for Decision Making

**DOI:** 10.3389/fpsyg.2021.705245

**Published:** 2021-11-03

**Authors:** Shahrina Md Nordin, Ammar Redza Ahmad Rizal, Izzal Asnira Zolkepli

**Affiliations:** ^1^Centre of Social Innovation, University of Technology Petronas, Tronoh, Malaysia; ^2^Faculty of Language and Communication, Universiti Malaysia Sarawak, Kota Samarahan, Malaysia; ^3^School of Communication, Universiti Sains Malaysia (USM), Penang, Malaysia

**Keywords:** social media, uses and gratifications theory, knowledge management, information quality, farmer, innovation diffusion, Facebook (FB)

## Abstract

Social media is a prominent communication platform. Its active usage permeates all generations and it is imperative that the platform be fully optimized for knowledge transfer and innovation diffusion. However, there are several considerations regarding platform usage, including media affordances. Social media affordances enable users to interact with the world around them through features of modality, agency, interactivity, and navigation. Previous studies have indicated that social media affordances significantly influence user behavior and usage. However, research exploring the effect of social media affordances on knowledge acquisition and the reduction of decision-making complexities is limited. Therefore, focusing on 179 paddy farmers in Malaysia, this study examined the effect of social media affordances on information quality, knowledge acquisition, and complexity reduction regarding innovation adoption decisions using a quantitative approach. This study’s findings reveal that social media affordances have a significant effect on perceived information quality, knowledge acquisition, and complexity reduction.

## Introduction

The flourishing popularity of social media has allowed its diffusion in all strata of society. At present, more than half (approximately 53.6%) of the global population uses at least one type of social media platform, including Facebook, Twitter, and Instagram (Smart Insight, 2021). With an average use of approximately two and a half (2.5) hours per day, social media has become an essential part of daily life, used mainly for information seeking (Smart Insight, 2021). The function of media as an information hub traces back to various communication or mass media models, such as the hypodermic model or the two-step model (Katz and Lazarsfeld, 1955; Bineham, 1988). Recently, the frequency of information-seeking behavior via social media has increased because it allows individuals from all backgrounds to share content or opinions without being limited by traditional mass media gatekeepers (Hargittai et al., 2018). This trend has gained prominence, particularly during the COVID-19 pandemic crisis, which began early 2020. Although the crisis has affected decision−making processes (Al Eid and Arnout, 2020), social media continues to grow significantly as the mainstream platform for communication in daily life (Saud et al., 2020).

Farmers make up one of the fast-emerging demographics that actively utilize social media to source information. Their access to information is crucial for agricultural development (Rogers, 2003; Mapiye et al., 2020). Conventionally, farmers rely on extension officers, peers, or mass media for information, particularly regarding new technology or innovation (Mannan et al., 2017; Listiana et al., 2020). However, with increasing internet accessibility, social media has gained importance among farmers as a channel to obtain information. Among other prominent social media applications, many farmers extensively used Facebook (Lee and Suzuki, 2020). Facebook capitalizes on its users’ networking capacity to promote content creation and information sharing (Kraus et al., 2019), attracting more users to the platform, and consequently increasing its user population. Diversity and inclusion affordances enable Facebook users to create groups based on similar interests or goals, allowing them to participate as group members. This phenomenon is known as virtual communities of practice (Wasko and Faraj, 2005; Lee and Suzuki, 2020) and farmers have become members of a variety of such communities, which they as information sharing and exchange platforms.

Nonetheless, farmers’ social media benefits remain limited. Current literature emphasizes the capability of social media as a new communication channel (Vannucci and McCauley Ohannessian, 2019) and general findings and discussions on social media as a tool for sourcing information benefit farmers by increasing their awareness of agricultural issues (Adnan et al., 2018). Other studies have examined the factors that affect the adoption of social media by the farmers (Ćirić et al., 2018), the purpose of using social media (Tao et al., 2020), and the motivation to use social media (Lee and Suzuki, 2020) for sharing information with other farmers.

Notwithstanding its revolutionary contribution to information sharing, the current findings from the literature seem to indicate that social media is rather similar to other conventional communication channels with the main intention being to disseminate information. However, although disseminating information is important, there is a need for researchers to focus on the mechanism through which social media provides new information to the farmers. Hence, to gain a new perspective, it is imperative for a study to answer the question of how social media disseminates information and what the properties or affordances of social media are that have an effect on the farmers, especially on their knowledge development.

Since the conception of mass media, scholars have developed an interest in exploring the motivation and purpose of its consumers’ behaviors (Katz et al., 1973). The phenomena described in a theoretical model known as the uses and gratification theory (U&G) was first to emphasize better comprehension of the individual or collective psychological factors that lead to media consumption and gratification (McQuail, 1984). This enabled scholars and media practitioners to develop effective approaches that improve media functionality to achieve their intended purposes (Blumler, 2019). The vast accessibility of the internet as well as rapid networking improvements have allowed an increase in internet users globally. As human beings strive to improve their connectivity to others, several social media have emerged. In this era of traditional social media, users are no longer considered “passive audiences,” (Sundar and Limperos, 2013); they actively participate in content creation, either for constructing, criticizing, or disseminating information amongst other users (Lofters et al., 2016). Furthermore, it has been argued that social media has redefined its current user roles from being a mere consumer to an active participant (Jung and Sundar, 2020).

This study addressed three research questions. First, do social media affordances significantly affect farmers’ perceived information quality? Second, does information quality in social media have a significant effect on farmers’ knowledge creation? Third, does acquired knowledge have a significant impact on complexity reduction for farmers? To address these research questions, we applied the U&G 2.0, whose theoretical construction was tailored to social media (Sundar and Limperos, 2013). Previous studies utilizing this theoretical perspective were mainly focused on users’ social media experiences (Wang et al., 2016), the role of emotions in social media use (Agur and Gan, 2021), its application to improve user experience (Ahn et al., 2021), and its effect on consumer activities (Flecha-Ortíz et al., 2021).

For this study, we considered Malaysian paddy farmers as an appropriate sample to meet the research objectives. The Malaysian paddy industry is regarded as the “sunset” industry, where its structure (Mannan et al., 2017) and heavy subsidies hinder innovation. It has been argued that farmers’ reliance on the subsidy prevents them from seeking better practices to improve their yield (Shamsudin et al., 2015). Therefore, understanding the effect of a Facebook group used by paddy farmers as a platform to seek information, debate, and discuss in the process of knowledge acquisition, is paramount.

The significance of our study is threefold. First, it expands the current model based on four classes of affordances—Modality, Agency, Interactivity, Navigability—identified as MAIN model in U&G 2.0 by Sundar and Limperos (2013), by introducing the role of a social structure as a source of gratification. Second, it links the role of social media affordances in affecting user knowledge acquisition through information quality, while addressing whether the knowledge acquired through social media (i.e., Facebook) has a significant effect on farmers’ agricultural development, particularly on reducing the complexities of innovation adoption. Finally, it expands on the mechanism through which social media affects farmers and agricultural development, a detail lacking in existing studies. Therefore, the findings from this study may benefit not only scholars in communication and innovation diffusion studies, but also practitioners or policymakers in enhancing agricultural development through social media.

## Background

Paddy is an important commodity in Malaysia, as rice is the staple food for the country. Its government allocates more than 300 million USD annually to subsidize paddy production (Mannan et al., 2017) distributed in the form of agricultural inputs, cash allocation, and other incentives, while imposing strict regulations to control the market (Arshad et al., 2019). Arguably, a highly subsidized and regulated market could hinder the farmers’ participation in knowledge creation due to over-reliance on government assistance.

**FIGURE 1:**
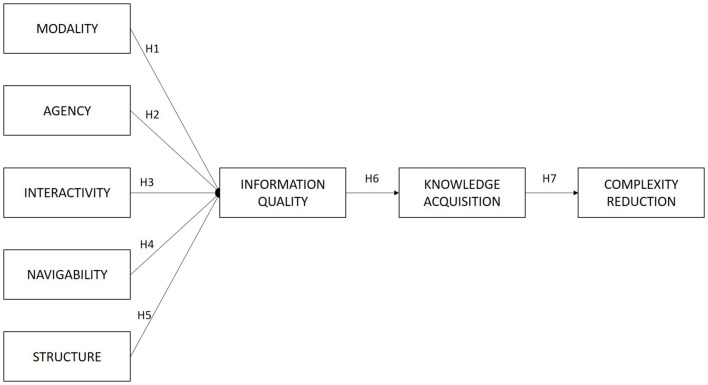
Theoretical framework of the study.

The introduction of social media has created a new environment in the Malaysian paddy industry. Farmers who previously relied heavily on local extension officers for sourcing information now turn to social media platforms. Among numerous social media platforms, Facebook is the most popular. Two Facebook groups, “*Padi Padi Padi*” and “*Padi oh Padi*,” are the most populous groups with a combined membership of more than 40,000 users. Figure 1 shows the front pages of both groups.

## Literature Review

### Uses and Gratification 2.0

There has been a shift in the framework of the U&G theory in recent decades. Until recently, prior research on U&G mainly suggested that an individual’s media use is dependent on their personal needs, particularly in traditional mass media. This argument is based on the original tenets of U&G where individual media consumption is deeply rooted in its psychological, and social needs (Katz et al., 1973). However, as technology advances and user participation on social media increases, gratification is no longer perceived as a psychological and social need of an individual. Currently, technology is considered a source of gratification that encourages users to continue using it (Wang et al., 2016). Lichtenstein and Rosenfeld (1983) were the first to explore media as a source of information. They found that gratification can also be based on various media features rather than the users’ needs. This is known as technological affordances. For instance, social media allows users to respond to content textually through semiotic expressions such as “Like” or “Heart” symbols on Facebook and Instagram, respectively. This interaction stimulates a person to engage continuously in social media platforms (Wang et al., 2016).

Therefore, the phenomena discovered in new forms of social media require an extension or modification from the traditional U&G theory. The continued expansion of features in social media demands a more nuanced approach to U&G theory. This limitation has brought several researchers to construct an improvised theoretical approach known as “U&G 2.0” (Sundar and Limperos, 2013; Jung and Sundar, 2020). The ability of modern media, including social media, to offer numerous affordances is a fundamental background favoring the development of the theory. U&G 2.0, is based on several technological affordances, including modality, agency, interactivity, and navigability—known as the MAIN model (Sundar and Limperos, 2013). These affordances were argued to have significant psychological consequences that trigger cognitive heuristics among users (Chen et al., 1999) while meeting some of their expectations.

### MAIN Model and Knowledge Acquisition

This study hypothesize that knowledge acquisition among farmers is undeniably related to the properties or affordances of a technology comprising a structure with multiple facets; thus, the combination of user-provided content and platform features creates an ideal structure to persuade users to gather information (Shao, 2009). The four affordances, modality, agency, interactivity, and navigation (Sundar and Limperos, 2013) were derived from the concept of cognitive heuristics. Sundar and Limperos (2013) asserted that it could either accelerate heuristic processing or guide systematic processing of content with more effort.

### Modality

The mode of delivering media that appeals to the user’s perceptual system is referred to as modality. Although identifiable in any media, through for example, visuals and audio, modality is distinct between traditional and modern media (Wang et al., 2016). The scope of social media combines text, audio, and visuals and includes animation, a unique characteristic reported to increase attention and perceptual bandwidth (Sundar and Limperos, 2013; Ahn et al., 2021). Modality influencing perception is attributable to the novel and realistic aspects of social media. Empirical studies have reported the ability of modality to affect user behavior. For instance, a study that investigated Pinterest found that novelty is a significant predictor of user intention to follow other users on a social media platform (Wang et al., 2016). For the present study, we assumed that affordances of modality in a Facebook group enable better information quality, leading to knowledge acquisition. Therefore, the first hypothesis of this study was:

**H1**: Modality in social media positively affects the farmers’ perceived information quality.

### Agency and Structure

The MAIN model describes agency as the second affordance on the internet that allows users to source information (Sundar and Limperos, 2013). The rise in new gratification has revolutionized the concept of information gatekeeping, previously controlled by designated media professionals such as editors, media owners, and senior executives. A previous study has shown that media users can be agentic and vested in their role as the source or sender of information (Sundar et al., 2012). Reasonably, the function of the agent as a source of gratification depends on heuristic development in the context of media. These heuristics, as products of social media use, maintain our intention to consume information through the media. For instance, in some cases, interaction among members on social networks, such as Facebook evolves into a discussion in which the platform enables its users to exchange comments (Lofters et al., 2016; Nijjer and Raj, 2020). These discussions are easily readable, leading to an availability heuristic among users, as they begin to appreciate the production of heuristics while exploring discussions, emerging heuristic demand patterns consequently promote media consumption (Jaspersen and Aseervatham, 2017).

However, user gratification from social media is not limited to agentic roles. Agentic roles strongly focus on the individual as the decision-maker; using social media depends entirely on the user’s consciousness. A previous study has shown that social media reflects the existing social structure of the physical world comprised in itself (Krämer and Conrad, 2017). As a social structure in the physical world can affect individual consciousness and action, a similar phenomenon can occur in the virtual world, specifically in social networks. Virtual interaction is determined not only by the need of an individual through their agency, but also through social structure elements such as norms and identity (Elder-Vass, 2010). For instance, active contribution in a Facebook group can enhance the individual status, the maintenance of which requires continued user participation. Therefore, considering that structure is a form of gratification that persuades the user to engage in social media, we hypothesized that it affects user information quality. Based on the presented arguments, the second and third hypotheses were:

**H2**: Agency in social media positively affect the farmers’ perceived information quality.

**H3**: Structure in social media positively affect the farmers’ perceived information quality.

### Interactivity and Navigation

The final two affordances of social media in the MAIN model are interactivity and navigation. Interactivity refers to the user’s ability to modify the content on social media in real time (Oh and Sundar, 2015; Wang et al., 2016). Simply put, interactivity is a revolutionary feature of technology that allows users to interact via social media platforms, a contrast to the traditional, static mass media (Sundar and Limperos, 2013). For instance, most of the social media tools allow users to respond textually to content published by other users including emotional expressions (e.g., “Like” button on Facebook) and redistribution of the content within a social network (e.g., “Retweet” feature on Twitter). Attributes associated with interactivity include interaction, activity, responsiveness, and dynamic control (Oh and Sundar, 2015). The mechanism of interactivity affecting cognitive function among users has been demonstrated by various empirical findings. A study conducted on the effect of interactivity of an application on health information found that different interactivity functions affect cognitive involvement and perceived active control, leading to health information recall (Pajor et al., 2020). The ability of interactivity to affect the user’s cognitive load and information recall is the main reason we hypothesized its ability to predict information quality. Therefore, the fourth hypothesis was:

**H4**: Interactivity positively affect the farmers’ perceived information quality.

Navigation is another important affordance of social media, which enables user activity on any platform (Sundar and Limperos, 2013). Browsing is an essential attribute of navigation affordance. Users have unlimited access to explore vast content compared to traditional media such as newspapers (Wang et al., 2016; Flecha-Ortíz et al., 2021). Furthermore, the browsing function confers users the authority to perform historical checks of the available content. For instance, searching for previously published content using the “search” function allows users the option to filter information such as by specific year—a feature missing in traditional media. During the traditional media era, users relied on archival sources such as video recordings, to retrieve past content. Hence, in addition to allowing users the freedom to browse, navigation affordance also enhances their capability to search for previous content. Therefore, users find a richer quality of information. Based on this argument, the fifth hypothesis was:

**H5**: Navigation positively affect the farmers’ perceived information quality.

### Information Quality and U&G 2.0

The advancement of new media with beneficial affordances has encouraged users to continue using it. In this study, we argued that farmers’ gratifications from their respective Facebook groups expose them to a plethora of information in social media. The ability of humans to actively seek, gather, and obtain information is key to their survival (Pirolli and Card, 1999). The notion of information quality is not new, especially in information systems (IS) and managerial decisions. In the IS literature, information quality is associated with the accuracy, convenience of access, and reliability of the information (Wiengarten et al., 2010). Other definitions describe information quality as completeness, conciseness, relevance, understandability, meaningfulness, timeliness, and comparability of information (DeLone and McLean, 1992; Wang and Liao, 2008). Despite these definitions, in the context of social media, information quality needs to be observed from the user’s perspective (Kuo and Lee, 2009); it must be relevant for their utilization.

As mentioned earlier, multiple affordances presented in the MAIN model attract users to remain engaged in social media. In this study, the farmers continued their participation in the Facebook group either as observers or as active participants. Heuristics is an important commodity in social media wherein it is continuously used and produced. This is based on the empirical evidences presented in the past studies where people’s behavior in social media are dependent on heuristic factors such as the professionalism of the website design (John et al., 2011), privacy disclosure behaviors of peers (Acquisti et al., 2015), and authentic visual heuristics (Brandimarte et al., 2013). Hence, most of the social media platforms utilize this condition to attract more users. Subsequently, the collection of heuristics underwent substantial cognitive function tests (Wang et al., 2020), following which the screening of what users considered quality information was conducted. This is a continuous process that can be achieved only through the U&G of the media, not as a one-time event. Furthermore, we sought to clarify that the quality of information affects knowledge acquisition. Therefore, the sixth hypothesis was:

**H6**: Information quality positively affect the farmers’ knowledge acquisition.

### Knowledge Acquisition and Complexity Reduction

Knowledge acquisition has been empirically proven to improve an individual’s performance in various corpora of literature (Nguyen et al., 2015; Zossou et al., 2020; Feroz et al., 2021). There are varied opinions regarding the knowledge-acquisition process. Scholars have claimed that knowledge can be acquired through individual experiences (Fauzi et al., 2019). Individual experiences of events usually generate tacit knowledge. Tacit knowledge is considered complex and difficult to articulate, as it is based on individual comprehension. Despite this, scholars in knowledge management have argued that tacit knowledge can be translated into explicit knowledge, which can be easily transferred and decoded (Fauzi et al., 2019; Feroz et al., 2021).

Initially, the process of transferring explicit knowledge can be achieved through individual interactions. Therefore, scholars have emphasized the role of teaching as pivotal to the knowledge transfer process (Fauzi et al., 2019). As pinnacles of knowledge, teachers are required to construct and guide the learning process for their students (Kember, 2009). In the case of farmers, extension officers primarily shoulder the teacher’s role (Ashoori et al., 2019). Extension officers, equipped with technical knowledge, must ideally interact and guide farmers regarding appropriate farming practices. However, in reality, extension officers face limitations, such as lack of capacity and time, which hinders the knowledge transfer process (Goldberger et al., 2015; Iskandar et al., 2020) and is detrimental to agricultural development.

Nevertheless, with the availability of social media platforms, farmers can acquire knowledge with ease. As discussed earlier, the use of social media contributes to the development of heuristic components that can transform into beneficial information upon substantial involvement of cognitive processes. However, it is essential to highlight that this process requires continuous usage or interaction with the social media platform, which has been strongly indicated by the potential of U&G 2.0. Knowledge acquisition by farmers through social media may prove beneficial for complexity reduction. Complexity is defined as *“*the degree to which an innovation is perceived as relatively difficult to understand and use” (Rogers, 2003, p. 257). Complexity has been considered a major barrier to new technology or practice adoption in agriculture (Lavoie et al., 2021). Most barriers are caused by farmers’ lack of understanding of new practices or technology. A previous study reported that the inability to comprehend technical aspects developed into intense frustration in farmers (Cahyono et al., 2020). Therefore, complexity reduction is an important aspect of both farmers’ and agricultural development. Hence, with the acquired knowledge complexity reduction for farmers can be achieved. Thus, the seventh hypothesis was:

**H7:** Knowledge acquisition positively affect the farmers’ complexity reduction.

The conceptual framework for this study, based on the developed hypotheses, is shown in Figure 1.

## Methodology

### Data and Sample

To test the hypotheses, we obtained data through an online survey. All study participants provided written informed consent prior to responding to the survey. Since the background of the study is based on farmers’ participation in Facebook groups, we identified two major Facebook groups related to paddy farming in Malaysia. The first is a public group known as “*Padi Padi Padi*” (Paddies), and the second is a private group known as “*Padi Oh Padi*” (Dear Paddy), with approximately 13,000 and 40,000 members, respectively.

To gain membership to these groups, a potential user must request permission from the groups’ administrators. We selected both groups due to their high memberships and large number of activities. On average, both Facebook groups have five new items published as “posts” daily, consisting of questions, sharing of experiences, expressing grievances, and promoting new products or technology. Although neither of these groups restricts the type of membership, the majority of active members (i.e., members who frequently post new content or actively respond to another post) are paddy farmers. Hence, the selection of these groups was based on the direction and objectives of this study.

For sample size determination, we conducted *a priori* analysis by using G-Power calculation software (Preacher and Hayes, 2004). The analysis allows sample sizes to be computed as functions of user-specified values for the required significance level α, the desired statistical power 1-β, and the to-be-detected population effect size. We determined that the total number of respondents required in this study was 132 respondents; based on the criteria of 0.1 minimum Cohen’s f^2^ effect size of 0.05, a critical *t*-value of 1.979, and a statistical power of 0.950 (Preacher and Hayes, 2004). To conduct the survey, electronic questionnaires were selected and developed by adopting and adapting items and concepts from several previous studies (Kuo and Lee, 2009; Wiengarten et al., 2010; Sundar and Limperos, 2013; Nguyen et al., 2015; Wang et al., 2016; Mannan et al., 2017; Xie et al., 2020).

The study was conducted in March 2021. We first contacted the administrators of the selected groups. Upon receiving their approval, a Facebook post containing a link was shared with both groups. Since our target population specifically required farmers as participants, the potential participants were first required to state their occupation. Those who reported farming as their occupation were granted access to the survey questions in this study. However, those who selected occupations other than farming were directed to the exit page of this survey. A second Facebook post was submitted 4 days after the first one and shared to ensure that we obtained the minimum required sample size. In total, there were 179 responses collected for use in the final analysis.

### Measures

All items, in each construct of the survey instrument, utilized a 5-point Likert scale for measurement. The list of all items is included in Supplementary Appendix A.

### Modality

Drawing from the works of Sundar and Limperos (2013) on improvised U&G 2.0, modality was used to measure different methods of presentation in social media. Modality includes the concepts of realism, openness, and novelty that can be appealing to the user’s perceptual system. There were six items in the questionnaire measuring these constructs, originally developed by Sundar and Limperos (2013) and Wang et al. (2016).

### Agency

Based on the findings of Wang et al. (2016) as well as Sundar and Limperos (2013), we developed a measurement for agency, which comprises six items, intended to measure how affordances of social media allow users to be the agent or sources of information (Sundar and Limperos, 2013).

### Interactivity

This construct was intended to measure how respondents perceived the social media platform—a Facebook group in this study—as well as how the platform contained and allowed interaction, activity, and dynamic control to its users. Interactivity is based on the concept that social media enables users to interact with and through the platform. Five (5) items in this construct were adapted from Sundar and Limperos (2013) in which it can be found in Supplementary Appendix A.

### Navigability

The last construct based on the MAIN model is intended to measure respondents’ perceptions of navigability affordance within the Facebook group. This includes aspects such as browsing and navigation aids. It consists of five items adapted from Sundar and Limperos (2013).

### Structure

The structure is an extension of the MAIN model. It is related to the role of a social structure as an essential affordance in explaining user’s media gratification. This construct comprises five items adapted from Sundar and Limperos (2013) and Ahmad Rizal (2020). Please refer to Supplementary Appendix A.

### Information Quality

This construct measures the participants’ perceptions of information quality from the social media platforms adapted from the instrument developed by Wiengarten et al. (2010) as well as Kuo and Lee (2009). It consists of four items and can be found in Supplementary Appendix A.

### Knowledge Acquisition

The knowledge acquisition component is measured by using four items adapted from Nguyen et al. (2015) and Feroz et al. (2021). These items measure the responses related to users acquiring knowledge from the relevant social media platform.

### Complexity Reduction

Complexity reduction is a construct that was developed based on Rogers (2003) diffusion of innovation theory. Four items measure users’ perceived reduction in the complexity of new technology or practices based on the information or knowledge obtained through social media. The items were adapted from Mannan et al. (2017).

### Pilot Study

A pilot study in which 30 farmers participated was conducted to test the instrument’s reliability and validity. The developed instrument was translated to Bahasa Melayu and back-translated to English to ensure its clarity and to convey what it is intended to measure (Sekaran and Bougie, 2015). The reliability scores of the instruments are shown in Table 1. All the constructs show a high Cronbach’s alpha score, indicating internal consistency. Thus, the instrument was used in this full-scale study. Apart from the reliability analysis, we ensured the validity of the instrument used in this study through face validity. The instrument was sent to two (2) expert panels in the field. No major changes were reported and the instrument was amended based on the panel’s suggestions. The final list of all instruments used in the study is included in Supplementary Appendix A.

**TABLE 1:** Cronbach’s alpha score for each construct.

Construct	Reliability (Cronbach’s alpha score)
Modality	0.854
Agency	0.813
Interactivity	0.779
Navigability	0.805
Structure	0.836
Perceived information quality	0.792
Knowledge acquisition	0.826
Complexity reduction	0.861

### Data Analysis

The collected responses were first screened for missing data. The data were then analyzed for demographic and descriptive findings using descriptive statistics. For this study, we used the Partial Least Square—Structural Equation Modeling (PLS-SEM) for inferential statistics and hypotheses testing (Hair et al., 2014; Henseler et al., 2016). Prior to the hypotheses testing, which comprises of structural model assessment, we conducted an evaluation of the measurement model needs to ensure the validity of the instrument used. There were three required assessments: internal consistency reliability, convergent validity, and discriminant validity (Ramayah et al., 2018). Findings from the first and second assessments are shown in Table 2, while the findings for the third assessment are included in the Supplementary Appendix B. All constructs show a composite reliability score between 0.7 and 0.9, indicating internal consistency and an average variance extraction (AVE) score of more than 0.5, indicating that the construct explains 50% of the variance of its item (Henseler et al., 2014). Additionally, the Heterotrait-Monotrait (HTMT) test was conducted to test the discriminant validity of the instrument, obtaining a score of less than 1 for each construct, indicating high discriminant validity (Ramayah et al., 2018).

**TABLE 2:** Cronbach’s alpha, composite reliability and average variance extraction (AVE) for each construct.

Construct	Composite reliability	Reliability (Cronbach’s alpha score)	Average variance extraction (AVE)
Modality	0.854	0.854	0.634
Agency	0.813	0.813	0.648
Interactivity	0.779	0.779	0.710
Navigability	0.805	0.805	0.658
Structure	0.836	0.836	0.762
Perceived information quality	0.792	0.792	0.688
Knowledge acquisition	0.826	0.826	0.709
Complexity reduction	0.861	0.861	0.655

## Results and Findings

This study engaged 179 farmers who are members of either the Facebook groups—“*Padi Oh Padi*” or “*Padi Padi Padi.*” The demographics of the respondents are presented in Table 3. To assess the hypotheses, we conducted an inferential analysis using PLS-SEM. The analysis required both measurement and structural models to pass the required assessment and to ensure the validity of the findings (Hair et al., 2019). There were six steps developed for assessing the integrity of the structural model (Hair et al., 2017): (1) Assess the structural model for collinearity, (2) assess the significance and relevance of the structural model relationship, (3) assess the level of R^2^, (4) assess the f^2^ effect size, (5) assess the predictive relevance, Q^2^, and (6) assess the q^2^ effect size. The details of the assessment findings are provided in the Supplementary Appendix C.

**TABLE 3:** Respondents’ profiles and demographics.

Respondent profiles	Frequency
**Respondents age**	
<25 years old	18
25–35 years old	52
36–45 years old	57
46–60 years old	41
>60 years old	11
**Education level**	
No formal education	5
Primary school	11
Secondary school	117
Post-secondary school (e.g., Technical Certificate, Diploma)	29
College degree (e.g., Bachelor’s Degree and above)	17
**Experience in paddy farming**	
<5 years	35
6–15 years	72
16–25 years	55
>25 years	17
**Social media usage (hours/day)**	
<1 h	51
1–3 h	82
3–5 h	31
>5 h	15

### Hypotheses Testing

Two different tests were conducted to test the hypotheses. However, prior to the hypothesis testing, to ensure the validity of the model tested in this study, we included age as the controlling factor and tested its relationship to information quality, knowledge acquisition, and complexity reduction. Based on the statistical analysis, it is reported that there is no significant effect between age and the three endogenous variables. The details for the value in this statistical analysis are included in Supplementary Appendix C.

For hypothesis testing, first, the predictive power of the relationship was measured using *R*^2^, to measure the variance explained in each endogenous construct (i.e., the dependent variable). The second test measured the statistical significance of each relationship through bootstrapping (Hair et al., 2019). A *p*-value of less than 0.05, and a T score of more than 1.96, is considered a statistically significant relationship; hence, the hypotheses are acceptable. The details of the findings are shown in Table 4. The findings indicate that agency, structure, and interactivity have a significant effect on information quality, and information quality has a significant effect on knowledge acquisition. Additionally, we also identified that knowledge acquisition has a significant effect on complexity reduction.

**TABLE 4:** Construct relationship and hypotheses testing.

	Std beta	T Statistics (| O/STDEV|)	*P*-values	Hypotheses testing
**H1**: Modality → perceived information quality	0.016	0.305	0.761	Not supported
**H2:** Agency → perceived information quality	0.283	9.431	<0.001	Supported
**H3:** Interactivity → perceived Information quality	0.165	3.054	<0.001	Supported
**H4:** Navigability → perceived information quality	0.042	1.072	0.524	Not supported
**H5:** Structure →perceived information quality	0.655	13.381	<0.001	Supported
**H6:** Perceived information quality →knowledge acquisition	0.267	5.052	<0.001	Supported
**H7:** Knowledge acquisition →complexity reduction	0.451	8.713	<0.001	Supported

## Discussion

### Affordances Influence Perceived Information Quality

The findings of this study show that affordances of social media affect the users’ information quality. Through statistical analysis, we identified that agency, structure, and interactivity in the Facebook groups had a significant effect on the farmers’ perception n of information quality, whereas modality and navigability did not.

In a previous study, it was reported that social media affordances and the users’ behavior during media use are interrelated (Wang et al., 2016). Multiple characteristics attract users to continue using it. This is the central argument of U&G 2.0, that the user’s gratification is not only centered on the audience, but also on the media itself (Sundar and Limperos, 2013). Moreover, Sundar and Limperos (2013) outlined that these phenomena can be explained by four affordances of the MAIN model. Several other studies have previously highlighted the significant capability of the MAIN model to explain user behavior, such as the study conducted by Wang et al. (2016) and another study on Snapchat by Flecha-Ortíz et al. (2021). Similarly, our study showed that several elements in the MAIN mode, namely agency, interactivity, and structure have an effect on the farmers as well. However, instead of affecting behavior, our study compliments these two previous studies where we show that the MAIN model does not only influence users’ behavior, but also their perceived information quality and knowledge acquisition.

The mechanism of how affordances can influence a user’s information quality could be explained by the interrelation between the user’s cognitive function and repetitive social media usage. In this study, interactivity allowed farmers to interact with any content shared by other users in the Facebook group. Constant virtual interaction with other users is one of the reasons for high social media usage (Wang et al., 2016; Vannucci and McCauley Ohannessian, 2019; Ahn et al., 2021). The frequent usage of social media exposes users to a variety of information. We believe that this is a necessary step in enabling the user to procure quality information. Similarly, other studies reported that reaction buttons found on Facebook increase the users’ engagement in social media (Matamoros-Fernández, 2018; Smoliarova et al., 2018).

Furthermore, both agency and structure are critical affordances in this study. The purpose of a Facebook group is to promote discussions on specific content or topics related to a common cause among members. Responding and engaging in virtual forum sessions benefits the users; by engaging in a dialog, users are not only involved in information sharing through discourse, but also in learning through others’ opinions. The capability of social media as a platform for learning agency has also been reported in other studies. For instance, Velasquez and Rojas (2017) found that social media users learn new political information through Facebook and Twitter. The interrelation between users through publishing and responding to content is a form of transferring meaning from one agency to another (Sundar and Limperos, 2013). These meanings are often encapsulated in the heuristic library, which is an essential product of social media interaction.

However, these meanings and heuristics are not necessarily based on agency alone. This study identified structure as a social media affordance that has a significant effect on information quality. This complements agency affordances, as proposed by Sundar and Limperos (2013). The vitality of the structure can also be found in another study on family health and Facebook in which researchers reported that leadership and trust play an important role in determining the success of the Facebook group (Lofters et al., 2016). Both leadership and trust are essential elements in enhancing society social structure (Giddens, 1984). Both elements act as a function in developing and redeveloping interrelation between actors in the society. As the number of relations in the society increase, it develops into several institutions. This institution facilitates information transfer from one actors to the another. The higher the trust of an actor on the institution, the better the information will be disseminated. For instance, school and teachers is an institution in society that is trusted to educate the kids or youngsters. Similarly, the structure in physical world is transferred into a virtual relation on social media where users trust the platform in disseminating quality information through the structure affordances.

### Knowledge Acquisition and Complexity Reduction

Findings from this study showed that information quality influences farmers’ knowledge acquisition and that knowledge acquisition has an effect on the farmer’s complexity reduction. The capability of information quality to affect knowledge acquisition relies on the production of heuristics. Social media enables both the utilization and reproduction of heuristics. Heuristics enable the formation of information quality, which facilitates knowledge acquisition. The ability of information quality to influence knowledge management or acquisition has also been reported in other studies (Wang and Liao, 2008; Kuo and Lee, 2009). Furthermore, this study identified the ability of knowledge acquired from the Facebook groups to reduce farmers’ complexity significantly. This finding supports that the formation of tacit or explicit knowledge could occur in a social media platform, substantiating the shift in farmers’ education from relying on extension officers to sourcing information on social media platforms. Moreover, the plethora of information and different people with various expertise found in the Facebook group justify these findings.

### The Effect on Farmers

On the other hand, findings from this study show that social media plays an important role in knowledge creation amongst farmers. Our findings extend the understanding of the mechanism through which social media interacts with farmers’ cognitive function. In a previous study, scholars reported that farmers are already active on social media platforms such as Facebook, YouTube, and Clubhouse in which the farmers and advisors are key generators and facilitators of knowledge flows (Goodman and Jaworska, 2020). Part of the main challenges amongst farmers is knowledge acquisition and knowledge transfer. Our study further shows that social media has the ability to influence new knowledge acquisition.

According to Rogers (2003), knowledge is an essential element to enhance innovation in a society. Thus, having the ability to procure knowledge through social media enables a better innovation culture inside the agricultural society. Our findings are similar to those of a study conducted in Ghana where they show that both extension officers and farmers use social media to disseminate information such as lectures as well as innovation and technical information (Munthali et al., 2021).

They further stated that interaction is essential, as information sharing requires an extensive number of users comprising both farmers and extension officers to use a social media platform. In comparison, our study shows that interactivity, which is an affordance that focuses on the interaction aspect, is vital and has a significant influence toward users’ (farmers) perceived information quality. Thus, both studies reiterate that information dissemination requires a well-equipped interaction through social media function as well as others utilizing the platform.

From the Malaysian farmers’ perspective, this study shows that farmers’ social media interaction is mirroring the actual farmer–farmer or farmer–extension officer interaction. Apart from the interaction play, an essential role in determining the farmers’ perceived information quality, the structure variable is also a significant influencer. The variable measures how the participant values social-structure-associated elements in social media. This further indicates that the Malaysian farmers imitate their reality and possibilities using them as a guide when interacting with social media, regardless of whether they possess a relationship with other users or not.

One of the fundamental factors that lead Malaysian farmers to value social structure is associated with their shared identity. Sharing the same language, demographic, and culture have been shown to have an effect on the farmers’ behavior in the other studies. For instance, in a study conducted among a small group of oil palm farmers in Malaysia, shared identity was a significant variable that influences the farmers’ decision to participate in sustainability initiatives (Ahmad Rizal et al., 2021). Thus, in our study, shared identity could also explain how structure is a significant social media affordance in influencing perceived information quality.

## Conclusion

The main motivation of this study was to address concerns about how social media affordances affect farmers’ knowledge acquisition, based on the current phenomenon of farmers using social media platforms, particularly those participating in Facebook groups. Drawing on the Malaysian paddy industry as the background and U&G as an overarching theoretical perspective, this study explored the effect of social media affordances on farmers’ perceived information quality, knowledge acquisition, and complexity reduction. It was identified that three social media affordances–interactivity, agency, and structure–are capable of affecting the information quality of farmers. The data suggested that information quality has an effect on knowledge acquisition, which in turn affects the farmers’ complexity reduction. Notably, this study enhances Sundar and Limperos’s (2013) MAIN model by differentiating the affordance of agency and structure, as well as explaining its capability.

However, there are a few limitations to this study. First, it does not assess the effect of social media content on information quality. Second, it does not investigate the differences between demographic factors such as age, gender, and location regarding knowledge acquisition among the farmers. Third, there are several other factors such as intrinsic motivation that can influence individual decision as well as complexity reduction (Deci and Ryan, 2002). Social media affordances might have an influence on these elements, though we do not include these variables in this study. These limitations should be addressed in future studies to strengthen the thesis of this study on the effect of social media affordances on complexity reduction.

Our findings could be a point of departure for two different directions. The first direction is to identify how social media affordances explain several other phenomena, such as different social identities across social media platforms and the construction, as well as the deconstruction of social structure through social media. The second direction is to focus on identifying other affordances currently found in the rich environment of social media. Although multiple studies have investigated several phenomena in social media, such as group identity (Chan, 2014), psychosocial well-being (Vannucci and McCauley Ohannessian, 2019), and social learning (Awidi et al., 2019), the development of social media affordances is still elementary. The rapid expansion of technology and the variety of newly introduced social media demands more research to be conducted in this area.

## Data Availability Statement

The original contributions presented in the study are included in the article/Supplementary Material, further inquiries can be directed to the corresponding author/s.

## Ethics Statement

Ethical review and approval was not required for the study on human participants in accordance with the local legislation and institutional requirements. The patients/participants provided their written informed consent to participate in this study.

## Author Contributions

SM: project administration, resources, supervision, data validation roles, writing—review, and editing. AA: conceptualization, data curation, data analysis, methodology, roles, writing—original draft. IZ: instrument review and editing, revision, data analysis, roles, writing—review, and editing. All authors contributed to the article and approved the submitted version.

## Conflict of Interest

The authors declare that the research was conducted in the absence of any commercial or financial relationships that could be construed as a potential conflict of interest.

## Publisher’s Note

All claims expressed in this article are solely those of the authors and do not necessarily represent those of their affiliated organizations, or those of the publisher, the editors and the reviewers. Any product that may be evaluated in this article, or claim that may be made by its manufacturer, is not guaranteed or endorsed by the publisher.
