# Supplementary material for: Innovation Diffusion: The Influence of Social Media Affordances on Complexity Reduction for Decision Making
Source: Front Psychol. 2021 Nov 3;12:705245. doi: 10.3389/fpsyg.2021.705245 (PMC8595103; doi:10.3389/fpsyg.2021.705245)
Supplement: Supplementary file 1 [file Table_1.DOCX]

**Appendix A – Items in Questionnaire and Factor Loading**

| Constructs | Items | Loadings |
| --- | --- | --- |
| Modality | I browse the Facebook group (group’s name) because I know the content is real and not made up | 0.730 |
|  | I browse the Facebook group (group’s name) because it is like communicating face to face | 0.838 |
|  | I browse the Facebook group (group’s name) because it creates the experience of being present in distant environments | 0.729 |
|  | I browse the Facebook group (group’s name) because the experience is very much like real life | 0.724 |
|  | I browse the Facebook group (group’s name) because the experience is unusual | 0.763 |
|  | I browse the Facebook group (group’s name) because the technology is innovative | 0.821 |
| Agency | I browse the Facebook group (group’s name) because it allows me to have my say | 0.852 |
|  | I browse the Facebook group (group’s name) because it allows me to send my thoughts to many others | 0.743 |
|  | I browse the Facebook group (group’s name) because it gives me the power to broadcast to the group’s member | 0.761 |
|  | I browse the Facebook group (group’s name) because it allows me to set my preferences | 0.805 |
|  | I browse the Facebook group (group’s name) because it features content that is a true reflection of myself | 0.822 |
|  | I browse the Facebook group (group’s name) because it allow me to share information with others | 0.774 |
| Interactivity | I browse the Facebook group (group’s name) because I feel active when I use it | 0.828 |
|  | I browse the Facebook group (group’s name) because it gives me control | 0.780 |
|  | I browse the Facebook group (group’s name) because I am able to control my interaction with others using the interface | 0.814 |
|  | I browse the Facebook group (group’s name) because I can specify my needs and preferences | 0.841 |
|  | I browse the Facebook group (group’s name) because I get to do a lot of things on it | 0.792 |
| Navigability | I browse the Facebook group (group’s name) because it allows me to obtain a wide variety of information | 0.819 |
|  | I browse the Facebook group (group’s name) because it allows me to browse freely | 0.772 |
|  | I browse the Facebook group (group’s name) because it is fun to explore | 0.810 |
|  | I browse the Facebook group (group’s name) because the interface helps me every step of the way | 0.718 |
|  | I browse the Facebook group (group’s name) because it allows me to surf for things that I am interested in | 0.883 |
| Structure | I browse the Facebook group (group’s name) because it allows me to expand my social network | 0.814 |
|  | I browse the Facebook group (group’s name) because I feel it is like my community | 0.863 |
|  | I browse the Facebook group (group’s name) because I believe the members of the groups will help me with my problems | 0.855 |
|  | I browse the Facebook group (group’s name) because members of the group use terms that I find is common | 0.776 |
|  | I browse the Facebook group (group’s name) because members of the groups share the same value amongst each other | 0.742 |
| Information Quality | By browsing the Facebook group (group’s name), I found the information shared is logical and reasonable | 0.817 |
|  | By browsing the Facebook group (group’s name), I found the information shared is meaningful | 0.838 |
|  | By browsing the Facebook group (group’s name), I found the information shared added value to my understanding | 0.807 |
|  | By browsing the Facebook group (group’s name), I found the information shared is available at a time suitable for its use. | 0.783 |
| Knowledge Acquisition | By browsing the Facebook group (group’s name), it allow me to acquire a new knowledge in paddy farming | 0.815 |
|  | By browsing the Facebook group (group’s name), it allow me to consistently acquire knowledge related to paddy farming | 0.798 |
|  | Interaction in the Facebook group (group’s name), allow me to obtain knowledge to solve my problems in paddy farming | 0.847 |
|  | By browsing the Facebook group (group’s name), it allow me to increase my knowledge related to paddy farming | 0.772 |
| Complexity Reduction | I found it is easier to use new technology after obtaining knowledge related to it from the Facebook group (group’s name) | 0.792 |
|  | By browsing the Facebook group (group’s name), the practices related to paddy farming that seems difficult before are becoming easier to learn | 0.867 |
|  | By browsing the Facebook group (group’s name), it helps me to understand more about new technology/practices related to paddy farming | 0.820 |
|  | I found that the content shared in the Facebook group (group’s name) are relevant for paddy farming | 0.809 |
