# Supplementary material for: Innovation Diffusion: The Influence of Social Media Affordances on Complexity Reduction for Decision Making
Source: Front Psychol. 2021 Nov 3;12:705245. doi: 10.3389/fpsyg.2021.705245 (PMC8595103; doi:10.3389/fpsyg.2021.705245)
Supplement: Supplementary file 2 [file Table_2.DOCX]

**Appendix B - Heterotrait-Monotrait ratio of correlation (HTMT) Result for Constructs in The Study**

|  | Modality | Agency | Interactivity | Navigability | Structure | Information  Quality | Knowledge Acquisition | Complexity Reduction |
| --- | --- | --- | --- | --- | --- | --- | --- | --- |
| Modality |  |  |  |  |  |  |  |  |
| Agency | 0.686 |  |  |  |  |  |  |  |
| Interactivity | 0.752 | 0.644 |  |  |  |  |  |  |
| Navigability | 0.723 | 0.587 | 0.734 |  |  |  |  |  |
| Structure | 0.612 | 0.724 | 0.672 | 0.596 |  |  |  |  |
| Information Quality | 0.641 | 0.629 | 0.714 | 0.707 | 0.651 |  |  |  |
| Knowledge Acquisition | 0.685 | 0.611 | 0.572 | 0.668 | 0.575 | 0.708 |  |  |
| Complexity Reduction | 0.621 | 0.624 | 0.664 | 0.571 | 0.511 | 0.688 | 0**.**652 |  |

Note: HTMT score of less than 1 indicate that there is discriminant validity amongst the constructs
