# Supplementary material for: Innovation Diffusion: The Influence of Social Media Affordances on Complexity Reduction for Decision Making
Source: Front Psychol. 2021 Nov 3;12:705245. doi: 10.3389/fpsyg.2021.705245 (PMC8595103; doi:10.3389/fpsyg.2021.705245)
Supplement: Supplementary file 3 [file Data_Sheet_1.DOCX]

**Appendix C – Structural Model Assessment of PLS-SEM**

Steps for assessment of structural model in PLS-SEM have been discussed in the last chapter. There are six (6) steps which are:

1. Assessment of structural model for collinearity issues
2. Assessment the significance and relevance of the structural model relationship
3. Assessment of the level of R^2^
4. Assessment of the Effect Size (f^2^)
5. Assessment of predictive relevance (Q^2^)
6. Assessment of the q^2^ effect size
7. **Assessment of structural model for collinearity issues**

Collinearity issue is important in validating structural model integrity as to avoid two sets of constructs to causally related (Ramayah, Cheah, Chuah, Ting, & Memon, 2018). Collinearity is measured through the variance inflation factor (VIF). A VIF value of 5 or higher indicates a potential collinearity problem (Sarstedt, Ringle, Smith, Reams, & Hair, 2014). All the inner VIF values of all sets of predictor constructs in the structural model are less than 5, indicating lateral multicollinearity is not a concern in the study and further examination of the model can be conducted. Table 1 shows the VIF values for each predictor constructs.

Table 1: Lateral Collinearity Assessments

| Construct | Modality | Agency | Interactivity | Navigability | Structure | Info.quality | Know.acq. | Comp.red. |
| --- | --- | --- | --- | --- | --- | --- | --- | --- |
| Modality |  | 1.257 | 1.257 | 1.679 | 1.174 | 1.368 | 1.291 | 1.027 |
| Agency |  |  | 1.474 | 1.682 | 1.453 | 1.582 | 1.124 | 1.338 |
| Interactivity |  |  |  | 1.714 | 1.692 | 1.740 | 1.628 | 1.284 |
| Navigability |  |  |  |  | 1.228 | 1.482 | 1.202 | 1.147 |
| Structure |  |  |  |  |  | 1.714 | 1.558 | 1.056 |
| Information  Quality |  |  |  |  |  |  | 1.951 | 1.472 |
| Knowledge acquisition |  |  |  |  |  |  |  | 1.357 |
| Complexity reduction |  |  |  |  |  |  |  |  |

1. **Assessment the significance and relevance of the structural model relationship**

The result of this assessment is shown in Table 3 in the main article.

1. **Assessment of the level of R^2^**

R^2^ is a measure of the model’s predictive accuracy and also can be viewed as a combined effect of exogenous variables on the endogenous variable (Ramayah et al., 2018). In general R^2^ values of 0.75, 0.50, or 0.25 for the endogenous construct can be described as respectively substantial, moderate, and weak (Hair, Hult, Ringle, & Sarstedt, 2017). Findings from the study were shown in Table 2.

Table 2: R^2^ Values for Endogenous Construct in The Study

| Constructs | R Square | R Square Adjusted |
| --- | --- | --- |
| Information Quality | 0.714 | 0.712 |
| Knowledge Acquisition | 0.590 | 0.584 |
| Complexity Reduction | 0.642 | 0.635 |

1. **Assessment of the Effect Size (f^2^)**

The effect size of the predictor constructs is evaluated using Cohen’s f^2^ (Wassertheil & Cohen, 1970). The effect size is based on the value of f^2^ where the value is considered large – 0.35, medium – 0.15, and 0.02 – small (Ramayah et al., 2018). Table 3 shows the f^2^ value found in the study.

Table 3: f^2^ Effect Size Found in The Study

| Construct | Information Quality | Knowledge Acquisition | Complexity Reduction |
| --- | --- | --- | --- |
| Modality | 0.008 |  |  |
| Agency | 0.215 |  |  |
| Interactivity | 0.148 |  |  |
| Navigability | 0.013 |  |  |
| Structure | 0.261 |  |  |
| Information  Quality |  | 0.283 |  |
| Knowledge acquisition |  |  | 0.336 |

1. **Assessment of predictive relevance (Q^2^)**

Predictive relevance (Q^2^) of the predictors in this study were assessed using the blindfolding procedure. If the resulting Q^2^ value is larger than 0, then it indicates that the exogenous constructs have predictive relevance for the endogenous construct under investigation (Fornell & Cha, 1994). Omission distance, D of 7 was used in the study. The study shows that all the three endogenous constructs have a value of:

- Information Quality – 0.597
- Knowledge Acquisition – 0.461
- Participative Behaviour – 0.518

The value thus indicating that the model has sufficient predictive relevance.

1. **Assessment of the q^2^ effect size**

The last step in assessing structural model in the study is the assessment of q^2^ effect size. It allows the assessment of the exogenous construct’s contribution to endogenous latent variable’s Q^2^ value. The value 0.35 and higher indicate strong effect, 0.15 and higher indicate moderate effect and value 0.02 and higher indicate a weak effect. Figure 1 below shows the formula in obtaining q^2^. Table 4 shows the q^2^ value found in this study.

$$q^{2}= \frac{Q^{2} included-Q^{2} excluded}{1-Q^{2} included}$$

Figure 1: Formula to Obtain q^2^ Effect Size

Table 4: q^2^ Value for The Study

| Exogenous/Endogenous Construct | Information Quality | Knowledge Acquisition | Complexity Reduction |
| --- | --- | --- | --- |
| Modality | 0.085 |  |  |
| Agency | 0.172 |  |  |
| Interactivity | 0.146 |  |  |
| Navigability | 0.075 |  |  |
| Structure | 0.247 |  |  |
| Information  Quality |  | 0.388 |  |
| Knowledge acquisition |  |  | 0.371 |

**REFERENCES**

Fornell, C., & Cha, J. (1994). Partial least squares. In *R. P. Bagozzi (Ed.), Advanced methods of marketing research*. https://doi.org/10.1007/978-3-319-05542-8

Hair, J. F., Hult, G. T. M., Ringle, C. M., & Sarstedt, M. (2017). *A primer on partial least squares structural equation modeling (PLS-SEM)* (Second Edi). Retrieved from https://uk.sagepub.com/en-gb/asi/a-primer-on-partial-least-squares-structural-equation-modeling-pls-sem/book244583

Ramayah, T., Cheah, J., Chuah, F., Ting, H., & Memon, M. A. (2018). Partial Least Squares Structural Equation Modeling (PLS-SEM) using SmartPLS 3.0: An update and Preactical Guide to Statistical Analysis. In *Handbook of Market Research*. https://doi.org/10.1213/01.ane.0000105862.78906.3d

Sarstedt, M., Ringle, C. M., Smith, D., Reams, R., & Hair, J. F. (2014). Partial least squares structural equation modeling ( PLS - SEM ) : A useful tool for family business researchers. *Journal of Family Business Strategy*. https://doi.org/10.1016/j.jfbs.2014.01.002

Wassertheil, S., & Cohen, J. (1970). Statistical Power Analysis for the Behavioral Sciences. *Biometrics*. https://doi.org/10.2307/2529115
